# Receptors and host factors: key players in human metapneumovirus infection

**DOI:** 10.3389/fcimb.2025.1557880

**Published:** 2025-04-01

**Authors:** Yingdong Dong, Zhengde Xie, Lili Xu

**Affiliations:** ^1^ Beijing Key Laboratory of Core Technologies for the Prevention and Treatment of Emerging Infectious Diseases in Children, National Clinical Research Center for Respiratory Diseases, National Key Discipline of Pediatrics (Capital Medical University), Beijing Research Center for Respiratory Infectious Diseases, Beijing Pediatric Research Institute, Beijing Children’s Hospital, Capital Medical University, National Center for Children’s Health, Beijing, China; ^2^ Research Unit of Critical Infection in Children, Chinese Academy of Medical Sciences, Beijing, China

**Keywords:** human metapneumovirus, receptors, host factors, virus-host interaction, noncoding RNAs

## Abstract

Human metapneumovirus (hMPV) is a significant global pathogen that causes acute respiratory tract infections, especially in infants, young children, the elderly, and immunocompromised individuals. Despite its increasing prevalence, there are currently no vaccines or effective treatments available for hMPV. The pathogenesis of hMPV infection is a complex process involving a multitude of host factors and viral receptors. These interactions determine the virus ability to enter host cells, replicate, and evade the immune response. This review is the first to provide a comprehensive overview of the current understanding of host–virus interactions in hMPV pathogenesis. By elucidating these mechanisms, we can identify potential targets for antiviral drugs and improve the management of hMPV infections.

## Introduction

Human metapneumovirus (hMPV) is an important cause of both upper and lower respiratory tract infections in infants and in elderly and immunocompromised patients worldwide (Falsey et al., 2003). Since its discovery in 2001, hMPV has been recognized as a major contributor to respiratory illnesses, ranging from mild upper respiratory tract disease to severe bronchiolitis and pneumonia. This results in substantial morbidity and mortality globally (van den Hoogen et al., 2001). Despite its clinical significance, there are currently no vaccines or specific antiviral treatments available for hMPV, highlighting the need for a deeper and more comprehensive understanding of the virus–host interactions that drive its pathogenesis.

HMPV is an enveloped, nonsegmented, negative-sense RNA virus that belongs to the *Pneumoviridae* family. Two major genotypes of the virus exist, hMPV-A and hMPV-B, with further subdivisions: A1, A2 (including the A2a, A2b, and A2c lineages), B1, and B2 (comprising the B2a and B2b lineages) (Piñana et al., 2023).

**Figure 1:**
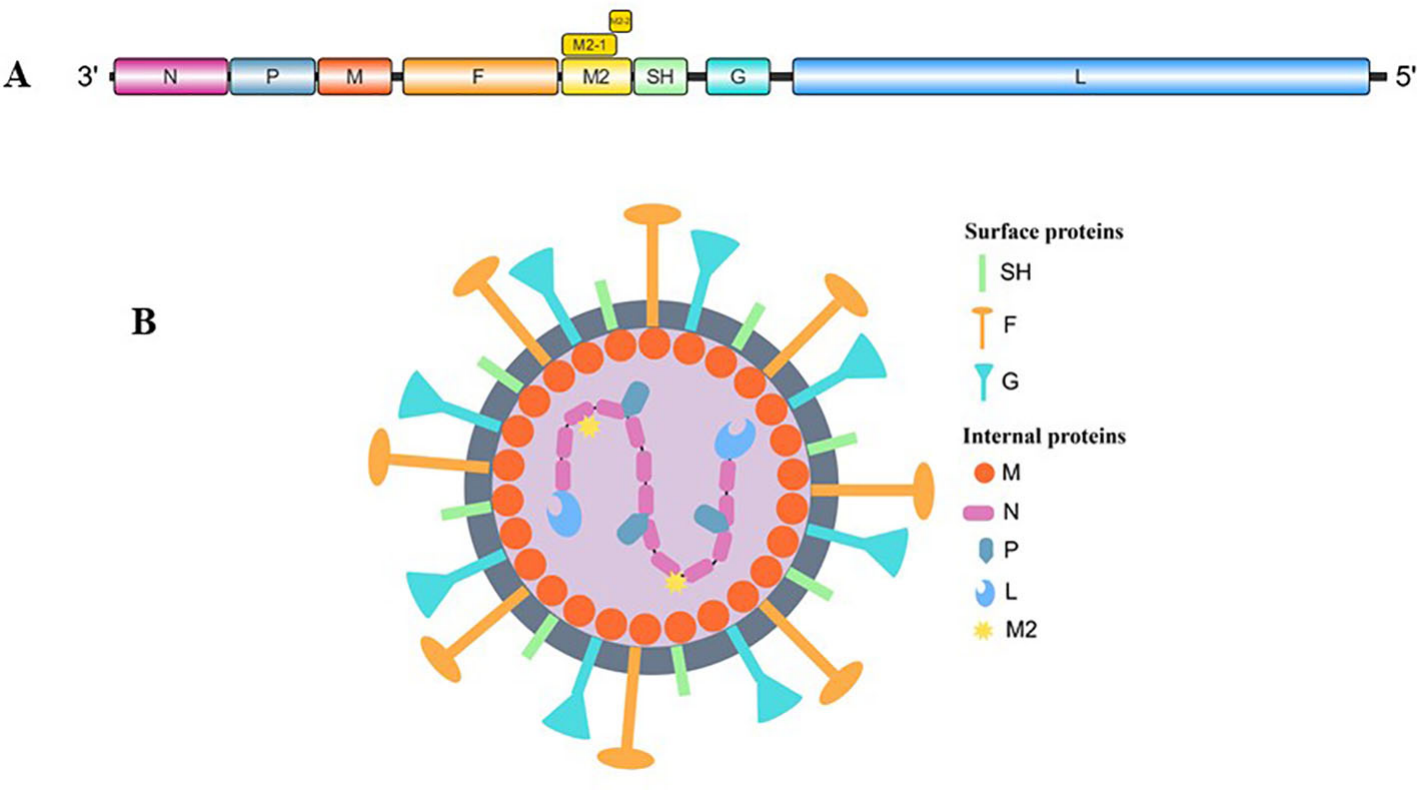
**(A)** Genomic organization of human metapneumovirus. **(B)** Schematic illustration of hMPV (By Figdraw).

The hMPV genome spans approximately 13 kb and consists of eight genes arranged in the sequence 3′-N-P-M-F-M2-SH-G-L-5′ (**Figure 1**), collectively encoding nine viral proteins. The M2 gene is unique because its mRNA contains two overlapping open reading frames (ORFs), producing M2-1 (putative transcription factor) and M2-2 (RNA synthesis regulatory factor) proteins (Biacchesi et al., 2007; Zhao et al., 2022). The virus expresses three membrane proteins crucial for host cell infection: F (fusion glycoprotein), G (attachment glycoprotein), and SH (small hydrophobic glycoprotein). The remaining viral components include N (nucleoprotein), P (phosphoprotein), M (matrix protein), and L (viral polymerase) (**Figure 1**) (van den Hoogen et al., 2002).

**Figure 2:**
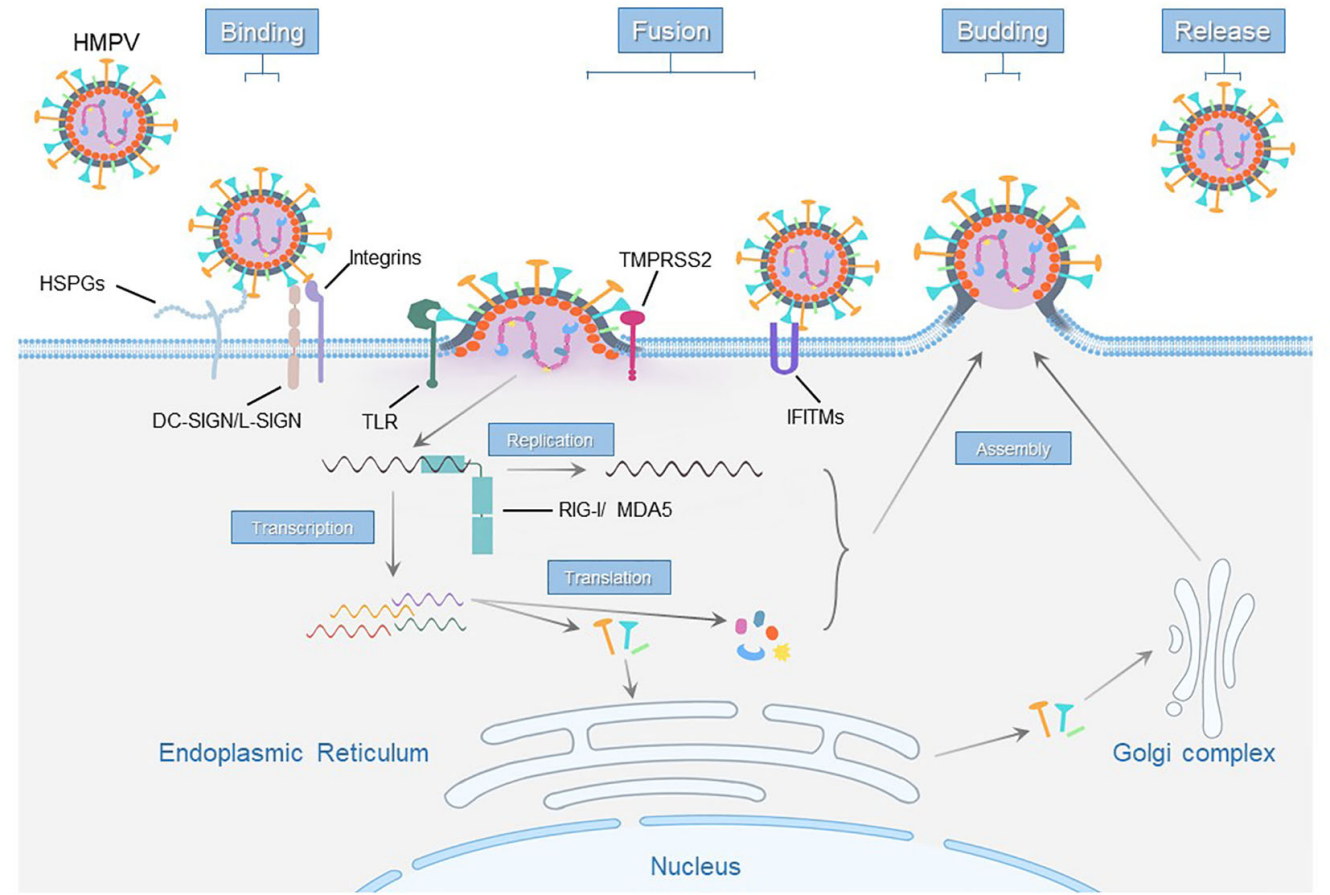
Schematic representation of host cell receptors and factors essential for the hMPV life cycle.

HMPV replication begins when the G and F proteins facilitate virus attachment and membrane fusion, facilitating the release of the RNA genome into the cell. The genome then undergoes transcription and replication, producing viral mRNAs and new genomes for protein synthesis. The newly synthesized viral genome combines with the P, N, L, and M2 proteins and becomes encased by the M protein. Concurrently, F, G, and SH proteins are modified in the endoplasmic reticulum and Golgi complex. These components then assemble on the cell membrane to form new virus particles, which bud from the cell surface (Schildgen et al., 2011; Feuillet et al., 2012; Panda et al., 2014) (**Figure 2**).

This review aims to provide a comprehensive overview of the current knowledge concerning the host factors, receptors and noncoding RNAs involved in hMPV infection. By elucidating the mechanisms by which hMPV interacts with host cells, we can contribute to the development of effective antiviral strategies and improve clinical outcomes for individuals affected by this pathogen.

## Receptors for hMPV infection

HMPV replicates extensively in airway epithelial cells (AECs) and persists there after completing its replication cycle (Kuiken et al., 2004; Soto et al., 2018). Previous studies revealed that infectious viruses were detectable in the lungs for up to 60 days. The most notable finding was the extraordinary persistence of viral genomic RNA, which remained detectable for as long as 180 days post infection, despite the presence of neutralizing antibodies (Alvarez et al., 2004). Although the precise mechanisms of hMPV immune evasion remain incompletely understood, multiple viral proteins have been shown to facilitate hMPV entry into host cells and evade immune responses (**Table 1**).

**Table 1:** Receptors for hMPV infection.

Receptors	Binding to	Functions	Cell/tissue distribution	References
HSPGs	G protein/F protein	promoting viral attachment and entry	ubiquitous expression	(Thammawat et al., 2008; Chang et al., 2012)
DC-SIGN/L-SIGN	G protein	promoting viral attachment and entry; mediate infection without GAGs	dendritic cells and endothelial cells	(Le Nouën et al., 2014; Gillespie et al., 2016)
Integrins	F protein	mediate viral fusion and entry; facilitate transcription and subsequent infection	multiple cell types	(Cseke et al., 2009; Cox et al., 2012)

The G protein of hMPV plays a crucial role in the initial stages of infection by interacting with heparan sulfate proteoglycans (HSPGs) (Thammawat et al., 2008; Chang et al., 2012), dendritic cell-specific intercellular adhesion molecule-3-grabbing nonintegrin (DC-SIGN) and liver/lymph node-specific intercellular adhesion molecule-3-grabbing nonintegrin (L-SIGN) (Le Nouën et al., 2014; Gillespie et al., 2016). These interactions facilitate viral attachment and entry into host cells. The F protein of hMPV mediates viral-host membrane fusion. Notably, this protein alone can facilitate both attachment and fusion in the absence of the G protein (Biacchesi et al., 2004; Cox and Williams, 2013). Integrins (Cseke et al., 2009; Cox et al., 2012) and HSPGs (Chang et al., 2012) have been implicated in the fusion and internalization processes mediated by the F protein of hMPV. The small hydrophobic (SH) protein of hMPV also contributes to the virus ability to evade the host immune response by inhibiting apoptosis in infected cells. Among these, HSPGs are important for hMPV attachment to cell surfaces because of their widespread expression and ability to bind to viral particles. The G and F proteins are involved in modulating the host immune response, aiding the virus in more effectively infecting host cells and evading immune surveillance. These functions enable hMPV to infect host cells more efficiently and replicate successfully.

### Heparan sulfate proteoglycans

Heparan sulfate proteoglycans (HSPGs) are widely expressed on the surface of various cell types and in the extracellular matrix (ECM). HSPGs consist of a core protein with one or more covalently attached heparan sulfate (HS) chains (Esko et al., 2009). HSPGs are multifunctional molecules that play vital roles in cell signalling, tissue organization, and disease progression. They play a significant role in viral infections by serving as an attachment and entry receptor for various viruses, such as respiratory syncytial virus (RSV) (Krusat and Streckert, 1997; Bourgeois et al., 1998), SARS-CoV-2 (De Pasquale et al., 2021), human immunodeficiency virus (HIV) (Bartlett and Park, 2011), and herpes simplex virus (HSV) (Shukla et al., 1999).

The G protein of hMPV has specific regions rich in positively charged amino acids that bind to the negatively charged HSPGs on the host cell surface. This interaction is crucial for the initial attachment and subsequent infection of host cells by hMPV, highlighting the G protein’s role as a key factor in the virus ability to infect and propagate within the host (Thammawat et al., 2008). The F protein is responsible for the fusion of the viral envelope with the host cell membrane, facilitating the entry of the virus into the host cell. This protein undergoes conformational changes that enable it to mediate membrane fusion at the cell surface or within endosomes. The binding of the F protein to HSPGs facilitates viral attachment and enhances infectivity (Chang et al., 2012). Although these two studies yielded different results, such discrepancy may be attributed to the distinct experimental approaches employed by different research groups: Chang et al. conducted experiments using G protein-deficient recombinant viruses (Chang et al., 2012), whereas Thammawat et al. primarily focused on characterizing the binding properties of recombinant G protein (Thammawat et al., 2008).

### Dendritic cell-specific intercellular adhesion molecule-3-grabbing nonintegrin and liver/lymph node-specific ICAM-3 grabbing nonintegrins

Dendritic cell-specific intercellular adhesion molecule-3-grabbing nonintegrin (DC-SIGN) is a type II membrane protein consisting of 404 amino acids with three distinct domains (Zhou et al., 2006). Its homolog, liver/lymph node-specific ICAM-3 grabbing nonintegrin (L-SIGN or DC-SIGNR), shares 73% identity with DC-SIGN at the nucleic acid level and has a similar genomic organization (Soilleux et al., 2000). These proteins exhibit distinct tissue-specific expression patterns. DC-SIGN is predominantly expressed on dendritic cells (DCs) within lymphoid tissues, mucosal surfaces, and the dermis. In contrast, L-SIGN is mainly found on endothelial cells, specifically in lymph nodes, placenta, and liver sinusoidal endothelial cells (LSECs). These distinct tissue distributions suggest different functional roles for these proteins across various anatomical sites (Lozach et al., 2007).

The extracellular domain consists of a stalk that mediates tetramerization and a COOH terminal carbohydrate recognition domain (CRD) that belongs to the C-type (Ca^2+^-dependent) lectin superfamily (Feinberg et al., 2001). This domain binds with high affinity to ICAM-3 (intercellular adhesion molecule 3), playing a role in pathogen recognition and immune response modulation (Geijtenbeek et al., 2000).

Although macropinocytosis has been identified as the primary route for hMPV entry into monocyte-derived dendritic cells (MDDCs), DC-SIGN-mediated endocytosis also plays a significant role as a supplementary pathway for hMPV entry and infection (Le Nouën et al., 2014). A study by Gillespie et al. demonstrated that both DC-SIGN and L-SIGN act as attachment factors for hMPV, facilitating viral binding to host cells independently of glycosaminoglycans (GAGs). These receptors enable hMPV infection through a calcium-dependent mechanism and a dynamin-mediated endocytosis pathway (Gillespie et al., 2016). These findings highlight the role of DC-SIGN and L-SIGN in facilitating alternative pathways for hMPV entry, broadening the understanding of viral infection mechanisms and identifying potential therapeutic targets.

### Integrins

Integrins, widely expressed across diverse cell types, are a family of cell adhesion receptors that are integral to a variety of cellular processes, including signal transduction, cell survival, proliferation, and migration. They are bidirectional signalling molecules that mediate cell–cell and cell–extracellular matrix interactions (Hynes, 2002). Integrins are heterodimers of noncovalently associated α and β subunits, each forming a single-pass type I transmembrane protein (Harburger and Calderwood, 2009). The α and β subunits have distinct domain structures, with extracellular domains from each subunit contributing to the ligand-binding site of the heterodimer. The sequence arginine-glycine-aspartic acid (RGD) was identified as a general integrin-binding motif, but individual integrins are also specific for particular protein ligands (Takada et al., 2007). These conformational shifts enable integrins to bind ligands and connect with the cytoskeleton and signalling pathways within the cell.

HMPV utilizes integrin receptors to facilitate its entry into host cells. Specifically, the hMPV fusion (F) protein contains an RGD motif, enabling it to interact with multiple RGD-binding integrins, such as αVβ1, αVβ5, αVβ6, and αVβ8 (Cox et al., 2012). Integrin αVβ1 consists of the αV (alpha V) and β1 (beta 1) subunits, which are required for efficient hMPV infection. Blockade of the cell surface αVβ1 integrin inhibits hMPV infectivity. Furthermore, the introduction of αV or β1 cDNAs into nonpermissive cells conferred hMPV infectivity, whereas the siRNA-mediated reduction in αV and β1 integrin expression inhibited hMPV infection (Cseke et al., 2009).

## Host restriction factors for hMPV infection

Host restriction factors are cellular proteins that constitute an essential component of the innate immune system, providing the first line of defence against viral infections. These proteins inhibit viral replication and dissemination within the host by targeting various stages of the viral life cycle. Host restriction factors are characterized by the following (1): Germ-Line Encoded: Most host restriction factors are encoded in the germ line and are present in almost all cell types. This ubiquity ensures a broad and rapid defence mechanism against a variety of viral pathogens (2). Interferon-Inducible and Constitutive Expression: Many restriction factors are inducible by interferons, a subset of which is constitutively expressed, allowing for immediate antiviral responses upon viral detection (3). Targeting Conserved Viral Components: These factors often target highly conserved viral components, making them effective against a wide range of viruses. This characteristic reduces the likelihood of viral escape through mutation (4). Additional Biological Functions: Besides their role in immunity, host restriction factors often have other biological functions, including cellular regulation, which may contribute to their antiviral activities (Kluge et al., 2015).

Host restriction factors are cellular proteins that inhibit viral replication and spread, playing crucial roles in antiviral defence. The key host restriction factors for hMPV include interferon-induced transmembrane proteins (IFITMs) (McMichael et al., 2018; Smith et al., 2019), retinoic acid-inducible gene I (RIG-I) (Bao et al., 2008; Liao et al., 2008), and melanoma differentiation-associated gene 5 (MDA5) (Ren et al., 2012; Baños-Lara Mdel et al., 2013; Spann et al., 2014) (**Table 2**).

**Table 2:** Host factors associated with hMPV infection.

Host factors	Functions	Cell/tissue distribution	References
Host restriction factors			
IFITMs	preventing viral entry and membrane fusion	ubiquitous expression	(McMichael et al., 2018; Smith et al., 2019)
RIG-I/MDA5	activating NF-κB and IRF transcription factors	ubiquitous expression	(Bao et al., 2008; Liao et al., 2008; Baños-Lara Mdel et al., 2013)
Host factors required for hMPV infection			
TLR4/7	reducing infiltration of inflammatory cells; facilitating viral entry; altering endosomal conditions	various immune cells	(Kolli et al., 2011; Velayutham et al., 2013; McMichael et al., 2018)
PAR-1	promoting viral replication	blood cells, epithelial cells and immune cells	(Lê et al., 2018)
TMPRSS2	facilitating viral fusion	epithelial cells	(Shirogane et al., 2008)

### Interferon-induced transmembrane proteins

Interferon-induced transmembrane proteins (IFITMs) are small transmembrane proteins that are ubiquitously expressed and characterized by conserved domains, including two hydrophobic regions that enable their integration into cellular membranes (Brass et al., 2009). At least three human IFITM proteins—IFITM1, IFITM2, and IFITM3—have antiviral activity; these proteins restrict early stages of replication in RSV (Everitt et al., 2013; Zhang et al., 2015), influenza A virus (Brass et al., 2009; Huang et al., 2011), flaviviruses (Brass et al., 2009; Jiang et al., 2010; Huang et al., 2011), SARS-CoV-2 (Prelli Bozzo et al., 2021), and others by inhibiting viral entry and fusion.

IFITM3 restricts endocytosed viral infections by blocking fusion and preventing viral genome entry into the cytosol, although the exact mechanism of inhibition remains unknown (Feeley et al., 2011). IFITM3 inhibits hMPV by blocking the virus fusion with host cell membranes via the hMPV F protein, and importantly, altering the level, localization, or activity of IFITM3 in cells can significantly affect hMPV infection (McMichael et al., 2018).

HMPV utilizes a bifurcated cellular entry strategy, either by fusing with the plasma membrane or with the endosomal membrane following endocytosis (Cox et al., 2015). IFITM3 is located predominantly in late endosomes and lysosomes, whereas IFITM1 is expressed mainly on the plasma membrane (Bailey et al., 2014; Weston et al., 2014). IFITM1 inhibits hMPV infection by blocking the fusion of the viral envelope with the host cell membrane, preventing the virus from entering the cytoplasm and establishing infection (Smith et al., 2019).

### Retinoic acid-inducible gene I and melanoma differentiation-associated gene 5

The RIG-I-like receptors (RLRs), expressed in most cell types, comprise retinoic acid-inducible gene I (RIG-I), melanoma differentiation-associated gene 5 (MDA5), and laboratory of genetics and physiology 2 (LGP2) play major roles in sensing RNA virus infections to initiate and modulate antiviral immunity (Loo and Gale, 2011). RLRs detect viral RNA or altered self-RNA within the cytoplasm, initiating innate immune responses and inflammatory processes, while also regulating gene expression to manage and counteract infections. RIG-I detects viral RNA generated during RNA virus replication, whereas MDA5 primarily recognizes synthetic dsRNA analogues such as poly(I:C) (Kato et al., 2006). The activation of RIG-I and MDA5 leads to the production of IFN and other inflammatory cytokines.

Silencing RIG-I using small interfering RNA (siRNA) or expressing dominant negative RIG-I mutants significantly decreases hMPV-induced expression of IFN-β, IL-8, and RANTES by inhibiting the activation of the NF-κB and IRF transcription factors, leading to increased viral replication (Bao et al., 2008; Liao et al., 2008). In hMPV-infected mice, the absence of MDA5 can increase the levels of CXCL1, IL-6, IL-1α, and G-CSF and lead to an increased inflammatory response and delayed recovery, as indicated by prolonged body weight loss and heightened pulmonary inflammation (Baños-Lara Mdel et al., 2013). Mitochondrial antiviral signalling protein (MAVS), also known as interferon-β promoter stimulator 1 (IPS-1), is crucial for the RLR pathway and facilitates the activation of NF-κB and IRF3, leading to the production of type I IFNs (Johnson and Gale, 2006; Seth et al., 2006).

## Host factors required for hMPV infection

Host factors can either promote or inhibit hMPV infection and replication. Several host-promoting factors facilitate hMPV infection and replication within the host. These factors increase the virus ability to infect cells, evade the immune system, and propagate within the host. Key promoting host factors have been reported for hMPV (**Table 2**): toll like receptor 4 (TLR4) (Kolli et al., 2011; Velayutham et al., 2013), toll like receptor 7 (TLR7) (McMichael et al., 2018), protease-activated receptor 1 (PAR-1) (Lê et al., 2018) and transmembrane protease, serine 2 (TMPRSS2) (Shirogane et al., 2008).

### Toll like receptors 4 and 7

To date, twelve members of the Toll-like receptor (TLR) family, predominantly expressed in immune cells, have been discovered in mammals. These TLRs are type I transmembrane glycoproteins that feature extracellular domains with varying numbers of leucine-rich-repeat (LRR) motifs. Additionally, they possess a cytoplasmic signalling domain known as the Toll/IL-1R homology (TIR) domain, which is similar to the domain found in the interleukin-1 receptor (IL-1R) (Bowie and O’Neill, 2000).

Based on their primary sequences, Toll-like receptors (TLRs) can be categorized into various subfamilies, each of which specializes in the recognition of specific pathogen-associated molecular patterns (PAMPs). TLR7, which recognizes nucleic acids, is localized within endosomes, whereas TLR4, which is capable of detecting a diverse array of ligands, is primarily found on the plasma membrane and can also localize to endosomes upon ligand binding (Akira et al., 2006; Kagan and Medzhitov, 2006).

TLR4 is crucial for both activating innate immune responses and contributing to disease pathology during hMPV infection. Compared with wild-type mice, mice lacking TLR4 exhibit significantly reduced inflammation and disease severity upon hMPV infection, as evidenced by less weight loss and airway obstruction. Silencing TLR4 expression significantly blocked hMPV-induced chemokine and type I interferon expression in both monocyte-derived dendritic cells (moDCs) and bone marrow-derived dendritic cells (BM-DCs). However, the ability to replicate and clear the virus, as well as T-cell proliferation and neutralizing antibody production, were unaffected (Kolli et al., 2011; Velayutham et al., 2013).

The presence of TLR7 leads to enhanced hMPV infection, revealing an unexpected role of this typically antiviral receptor. TLR7 and IFITM3 are both endosomal proteins that have opposing effects on hMPV infection, with TLR7 promoting infection and IFITM3 restricting it. Additionally, HEK293T cells coexpressing TLR7 and IFITM3 presented altered infection rates, suggesting a complex relationship in which TLR7 can modulate the host antiviral response, potentially impacting the overall dynamics of hMPV infection (McMichael et al., 2018).

### Protease-activated receptor 1

Protease-activated receptor 1 (PAR-1) is a member of the G protein-coupled receptor (GPCR) family that is activated by proteolytic cleavage, particularly by the enzyme thrombin. This 425-amino-acid receptor contains seven transmembrane-spanning domains and a thrombin cleavage site between residues 41 and 42 (Sidhu et al., 2014). PAR-1 is widely distributed, with expression in all blood cell types as well as epithelial and immune cells (Liu et al., 2017). Upon activation, PAR-1 mediates various cellular responses, including platelet aggregation, endothelial cell activation, and inflammation (Coughlin, 2000).

Inhibiting PAR-1 with the antagonist RWJ-56110 effectively reduced both viral replication and inflammation associated with hMPV infection. Specifically, the inhibition of PAR-1 led to decreased weight loss and mortality in infected mice, highlighting its detrimental role in these viral infections (Lê et al., 2018).

### Transmembrane protease, serine 2

Transmembrane protease, serine 2 (TMPRSS2) is a type II transmembrane protein with intracellular, LDL receptor class A (LDLRA), scavenger receptor cysteine-rich (SRCR), and serine protease domains (Paoloni-Giacobino et al., 1997). This protease is predominantly expressed in epithelial cells and shows androgen-dependent upregulation in prostate cancer (Lin et al., 1999; Afar et al., 2001). Functionally, TMPRSS2 plays a crucial role in the infection process of various respiratory viruses, such as SARS-CoV-2 (Hoffmann et al., 2020) and influenza viruses (Böttcher et al., 2006).

The generation of Vero cells that constitutively express TMPRSS2 greatly enhances the cleavage and activation of the HMPV F protein, which is crucial for the fusion of the viral membrane with the host cell membrane. This efficient cleavage and activation of the F protein by TMPRSS2 significantly improves the replication and multiplication of HMPV in host cells (Shirogane et al., 2008).

## Noncoding RNAs

Noncoding RNAs (ncRNAs) are functional RNA molecules that play regulatory roles without participating in protein synthesis. NcRNAs are categorized into two main groups on the basis of their functions: housekeeping and regulatory ncRNAs. Regulatory ncRNAs are subdivided according to their length. Short regulatory ncRNAs (sncRNAs, <200 nucleotides) include PIWI-interacting RNAs and microRNAs (miRNAs). The long ncRNAs (lncRNAs, ≥500 nucleotides) include long intergenic noncoding RNAs (lincRNAs), pseudogenic RNAs (PGs), natural antisense transcripts (NATs), and circular RNAs (circRNAs) (Eddy, 2001; Poliseno et al., 2024).

MiRNAs are (19–24 nucleotides) regulators found in animals, plants, and viruses. They bind to complementary sequences in the 3′ UTRs of target mRNAs to repress translation and/or trigger mRNA degradation (Skalsky and Cullen, 2010; George et al., 2024). MiRNAs can have dual effects on viral replication. Some miRNAs show inhibitory effects, such as miR-24 and miR-93, which target vesicular stomatitis virus (VSV) RNA and promote antiviral defence (Otsuka et al., 2007). Others have demonstrated promotional effects, as exemplified by miR-146a, which enhances Hendra virus (HeV) replication by suppressing RNF11 (Stewart et al., 2013).

Analysis of hMPV infection revealed altered cellular expression profiles, with miRNA levels increasing from 66.51% to over 86% after 15 hours. Among these changes, 142 miRNAs were upregulated, and 32 were downregulated (Deng et al., 2014). Notably, let-7f was significantly upregulated and exhibited antiviral effects: its inhibitors increased viral replication, whereas its mimics reduced it. The viral M2-2 protein regulated miRNAs such as miR-16 and miR-30a: miR-16 regulation depended on type I IFN signalling, whereas miR-30a was IFN independent, suggesting potential therapeutic targets (Wu et al., 2020).

Furthermore, hsa-miR-4634 enhances viral immune evasion by inhibiting type I interferon responses and interferon-stimulated genes, increasing viral replication in macrophages and epithelial cells (Martínez-Espinoza et al., 2023).

## Prospect

Since its discovery in 2001, hMPV has emerged as the second leading cause of bronchiolitis and pneumonia in children under 5 years of age. Despite its significant impact, no specific antiviral treatments or vaccines are currently available. Similar to the hRSV vaccine, formalin or heatinactivated hMPV vaccines led to a low induction of neutralising antibodies and enhanced immunemediated respiratory diseases in animal models (Hamelin et al., 2007; Yim et al., 2007). However, promising candidates are now in the preclinical research phase, offering hope for future prevention and treatment options. Notably, two vaccine candidates, one based on mRNAs and the other on virus-like particles, have progressed to phase I clinical trials (NCT05664334 and NCT05743881) (Guo et al., 2023). Furthermore, two additional candidates have already been evaluated in phase I clinical trials. A live attenuated vaccine (NCT01255410) was discontinued due to low infectivity and immunogenicity in seronegative children (Karron et al., 2018). Moreover, a combination mRNA vaccine (NCT03392389) targeting both hMPV and parainfluenza virus type 3 (PIV3) has shown promising safety and tolerability in healthy adults, whereas protective antibody levels for both hMPV and PIV3 are unknown (August et al., 2022). Neutralising monoclonal antibodies (nMAbs) are the core effectors of vaccines and are essential therapeutic immune drugs against infectious pathogens. The development of nMAbs against hMPV has accelerated in recent years as a result of breakthroughs in viral fusion (F) protein structural biology and experience with hRSV and other enveloped viruses (Guo et al., 2023). Among them, fully human antibodies, representing the predominant class (51%) of therapeutic monoclonal antibodies, have gained significant attention due to their low immunogenicity and excellent safety profile (Lu et al., 2020). To date, over 600 monoclonal antibodies (mAbs) against hMPV have been developed, with the majority being fully human antibodies derived from approximately 50 donors. These mAbs exhibit potent binding affinity and demonstrate broad-spectrum neutralizing capabilities (Guo et al., 2023). Furthermore, a study has shown that the incorporation of RSV F protein epitopes into cold-adapted influenza vaccines provides effective protection against RSV infection (Xu et al., 2024). Considering the similar respiratory tropism and infection patterns observed between hMPV and RSV, the incorporation of hMPV F protein epitopes into cold-adapted influenza vaccine platforms may represent a rational strategy.

Insufficient knowledge of how hMPV enters cells and interacts with the immune system has greatly hindered the development of vaccines and treatments for this virus. Here, we summarize the receptors, host factors and ncRNAs currently associated with hMPV infection (**Figure 2**), providing valuable insights into this significant respiratory pathogen. This information is crucial for developing targeted therapeutic strategies, improving our ability to combat hMPV, and offering prospective vaccines and treatments.

Currently, despite extensive efforts by researchers, the precise mechanisms of hMPV receptor interactions and host factor functions remain incompletely understood, and their expression regulation under pathological conditions is yet to be elucidated. Among the aforementioned factors, HSPGs serve as dual-ligand attachment factors in hMPV infection, interacting with both the viral G and F proteins during entry, making them attractive therapeutic targets. The extensive presence of HSPGs across tissues indicates their possible therapeutic applications. Although modifying HSPG functions raises concerns about cell stability, molecules designed to interrupt virus–HSPG binding may represent promising treatment strategies. Comprehensive research is needed to validate this therapeutic approach.

## References

[B1] Afar D. E. Vivanco I. Hubert R. S. Kuo J. Chen E. Saffran D. C.. (2001). Catalytic cleavage of the androgen-regulated TMPRSS2 protease results in its secretion by prostate and prostate cancer epithelia. Cancer Res. 61, 1686–1692. 11245484

[B2] Akira S. Uematsu S. Takeuchi O. (2006). Pathogen recognition and innate immunity. Cell 124, 783–801. doi: 10.1016/j.cell.2006.02.015 16497588

[B3] Alvarez R. Harrod K. S. Shieh W. J. Zaki S. Tripp R. A. (2004). Human metapneumovirus persists in BALB/c mice despite the presence of neutralizing antibodies. J. Virol. 78, 14003–14011. doi: 10.1128/JVI.78.24.14003-14011.2004 15564507 PMC533920

[B4] August A. Shaw C. A. Lee H. Knightly C. KalidIndia S. Chu L.. (2022). Safety and immunogenicity of an mRNA-based human metapneumovirus and parainfluenza virus type 3 combined vaccine in healthy adults. Open Forum Infect. Dis. 9, ofac206. doi: 10.1093/ofid/ofac206 35794943 PMC9251669

[B5] Bailey C. C. Zhong G. Huang I. C. Farzan M. (2014). IFITM-family proteins: the cell’s first line of antiviral defense. Annu. Rev. Virol. 1, 261–283. doi: 10.1146/annurev-virology-031413-085537 25599080 PMC4295558

[B6] Baños-Lara Mdel R. Ghosh A. Guerrero-Plata A. (2013). Critical role of MDA5 in the interferon response induced by human metapneumovirus infection in dendritic cells and in *vivo* . J. Virol. 87, 1242–1251. doi: 10.1128/JVI.01213-12 23152520 PMC3554051

[B7] Bao X. Liu T. Shan Y. Li K. Garofalo R. P. Casola A. (2008). Human metapneumovirus glycoprotein G inhibits innate immune responses. PloS Pathog. 4, e1000077. doi: 10.1371/journal.ppat.1000077 18516301 PMC2386556

[B8] Bartlett A. H. Park P. W. (2011). Heparan sulfate proteoglycans in infection. Glycans Dis. Ther., 31–62. doi: 10.1007/978-3-642-16833-8_2

[B9] Biacchesi S. Murphy B. R. Collins P. L. Buchholz U. J. (2007). Frequent frameshift and point mutations in the SH gene of human metapneumovirus passaged in *vitro* . J. Virol. 81, 6057–6067. doi: 10.1128/JVI.00128-07 17376897 PMC1900297

[B10] Biacchesi S. Skiadopoulos M. H. Yang L. Lamirande E. W. Tran K. C. Murphy B. R.. (2004). Recombinant human Metapneumovirus lacking the small hydrophobic SH and/or attachment G glycoprotein: deletion of G yields a promising vaccine candidate. J. Virol. 78, 12877–12887. doi: 10.1128/JVI.78.23.12877-12887.2004 15542640 PMC525014

[B11] Böttcher E. Matrosovich T. Beyerle M. Klenk H. D. Garten W. Matrosovich M. (2006). Proteolytic activation of influenza viruses by serine proteases TMPRSS2 and HAT from human airway epithelium. J. Virol. 80, 9896–9898. doi: 10.1128/JVI.01118-06 16973594 PMC1617224

[B12] Bourgeois C. Bour J. B. Lidholt K. Gauthray C. Pothier P. (1998). Heparin-like structures on respiratory syncytial virus are involved in its infectivity in *vitro* . J. Virol. 72, 7221–7227. doi: 10.1128/JVI.72.9.7221-7227.1998 9696816 PMC109944

[B13] Bowie A. O’Neill L. A. (2000). The interleukin-1 receptor/Toll-like receptor superfamily: signal generators for pro-inflammatory interleukins and microbial products. J. Leukoc. Biol. 67, 508–514. doi: 10.1002/jlb.67.4.508 10770283

[B14] Brass A. L. Huang I. C. Benita Y. John S. P. Krishnan M. N. Feeley E. M.. (2009). The IFITM proteins mediate cellular resistance to influenza A H1N1 virus, West Nile virus, and dengue virus. Cell 139, 1243–1254. doi: 10.1016/j.cell.2009.12.017 20064371 PMC2824905

[B15] Chang A. Masante C. Buchholz U. J. Dutch R. E. (2012). Human metapneumovirus (HMPV) binding and infection are mediated by interactions between the HMPV fusion protein and heparan sulfate. J. Virol. 86, 3230–3243. doi: 10.1128/JVI.06706-11 22238303 PMC3302303

[B16] Coughlin S. R. (2000). Thrombin signalling and protease-activated receptors. Nature 407, 258–264. doi: 10.1038/35025229 11001069

[B17] Cox R. G. Livesay S. B. Johnson M. Ohi M. D. Williams J. V. (2012). The human metapneumovirus fusion protein mediates entry via an interaction with RGD-binding integrins. J. Virol. 86, 12148–12160. doi: 10.1128/JVI.01133-12 22933271 PMC3486500

[B18] Cox R. G. Mainou B. A. Johnson M. Hastings A. K. Schuster J. E. Dermody T. S.. (2015). Human metapneumovirus is capable of entering cells by fusion with endosomal membranes. PloS Pathog. 11, e1005303. doi: 10.1371/journal.ppat.1005303 26629703 PMC4667933

[B19] Cox R. G. Williams J. V. (2013). Breaking in: human metapneumovirus fusion and entry. Viruses 5, 192–210. doi: 10.3390/v5010192 23325326 PMC3564117

[B20] Cseke G. Maginnis M. S. Cox R. G. Tollefson S. J. Podsiad A. B. Wright D. W.. (2009). Integrin alphavbeta1 promotes infection by human metapneumovirus. Proc. Natl. Acad. Sci. U.S.A. 106, 1566–1571. doi: 10.1073/pnas.0801433106 19164533 PMC2629439

[B21] Deng J. Ptashkin R. N. Wang Q. Liu G. Zhang G. Lee I.. (2014). Human metapneumovirus infection induces significant changes in small noncoding RNA expression in airway epithelial cells. Mol. Ther. Nucleic Acids 3, e163. doi: 10.1038/mtna.2014.18 24845106 PMC4040629

[B22] De Pasquale V. Quiccione M. S. Tafuri S. Avallone L. Pavone L. M. (2021). Heparan sulfate proteoglycans in viral infection and treatment: A special focus on SARS-coV-2. Int. J. Mol. Sci. 22(12):6574. doi: 10.3390/ijms22126574 34207476 PMC8235362

[B23] Eddy S. R. (2001). Non-coding RNA genes and the modern RNA world. Nat. Rev. Genet. 2, 919–929. doi: 10.1038/35103511 11733745

[B24] Esko J. D. Kimata K. Lindahl U. (2009). “Proteoglycans and sulfated glycosaminoglycans,” in Essentials of glycobiology, 2nd edition Cold Spring Harbor, NY: Spring Harbor Laboratory Press. 20301236

[B25] Everitt A. R. Clare S. McDonald J. U. Kane L. Harcourt K. Ahras M.. (2013). Defining the range of pathogens susceptible to Ifitm3 restriction using a knockout mouse model. PloS One 8, e80723. doi: 10.1371/journal.pone.0080723 24278312 PMC3836756

[B26] Falsey A. R. Erdman D. Anderson L. J. Walsh E. E. (2003). Human metapneumovirus infections in young and elderly adults. J. Infect. Dis. 187, 785–790. doi: 10.1086/jid.2003.187.issue-5 12599052

[B27] Feeley E. M. Sims J. S. John S. P. Chin C. R. Pertel T. Chen L. M.. (2011). IFITM3 inhibits influenza A virus infection by preventing cytosolic entry. PloS Pathog. 7, e1002337. doi: 10.1371/journal.ppat.1002337 22046135 PMC3203188

[B28] Feinberg H. Mitchell D. A. Drickamer K. Weis W. I. (2001). Structural basis for selective recognition of oligosaccharides by DC-SIGN and DC-SIGNR. Science 294, 2163–2166. doi: 10.1126/science.1066371 11739956

[B29] Feuillet F. Lina B. Rosa-Calatrava M. Boivin G. (2012). Ten years of human metapneumovirus research. J. Clin. Virol. 53, 97–105. doi: 10.1016/j.jcv.2011.10.002 22074934

[B30] Geijtenbeek T. B. Torensma R. van Vliet S. J. van Duijnhoven G. C. Adema G. J. van Kooyk Y.. (2000). Identification of DC-SIGN, a novel dendritic cell-specific ICAM-3 receptor that supports primary immune responses. Cell 100, 575–585. doi: 10.1016/S0092-8674(00)80693-5 10721994

[B31] George T. P. Subramanian S. Supriya M. H. (2024). A brief review of noncoding RNA. Egyptian J. Med. Hum. Genet. 25, 98. doi: 10.1186/s43042-024-00553-y

[B32] Gillespie L. Gerstenberg K. Ana-Sosa-Batiz F. Parsons M. S. Farrukee R. Krabbe M.. (2016). DC-SIGN and L-SIGN are attachment factors that promote infection of target cells by human metapneumovirus in the presence or absence of cellular glycosaminoglycans. J. Virol. 90, 7848–7863. doi: 10.1128/JVI.00537-16 27334579 PMC4988148

[B33] Guo L. Li L. Liu L. Zhang T. Sun M. (2023). Neutralising antibodies against human metapneumovirus. Lancet Microbe 4, e732–e744. doi: 10.1016/S2666-5247(23)00134-9 37499668

[B34] Hamelin M. E. Couture C. Sackett M. K. Boivin G. (2007). Enhanced lung disease and Th2 response following human metapneumovirus infection in mice immunized with the inactivated virus. J. Gen. Virol. 88, 3391–3400. doi: 10.1099/vir.0.83250-0 18024909

[B35] Harburger D. S. Calderwood D. A. (2009). Integrin signalling at a glance. J. Cell Sci. 122, 159–163. doi: 10.1242/jcs.018093 19118207 PMC2714413

[B36] Hoffmann M. Kleine-Weber H. Pöhlmann S. (2020). A multibasic cleavage site in the spike protein of SARS-coV-2 is essential for infection of human lung cells. Mol. Cell 78, 779–784.e5. doi: 10.1016/j.molcel.2020.04.022 32362314 PMC7194065

[B37] Huang I. C. Bailey C. C. Weyer J. L. Radoshitzky S. R. Becker M. M. Chiang J. J.. (2011). Distinct patterns of IFITM-mediated restriction of filoviruses, SARS coronavirus, and influenza A virus. PloS Pathog. 7, e1001258. doi: 10.1371/journal.ppat.1001258 21253575 PMC3017121

[B38] Hynes R. O. (2002). Integrins: bidirectional, allosteric signaling machines. cell 110, 673–687. doi: 10.1016/S0092-8674(02)00971-6 12297042

[B39] Jiang D. Weidner J. M. Qing M. Pan X. B. Guo H. Xu C.. (2010). Identification of five interferon-induced cellular proteins that inhibit west nile virus and dengue virus infections. J. Virol. 84, 8332–8341. doi: 10.1128/JVI.02199-09 20534863 PMC2916517

[B40] Johnson C. L. Gale M. Jr. (2006). CARD games between virus and host get a new player. Trends Immunol. 27, 1–4. doi: 10.1016/j.it.2005.11.004 16309964

[B41] Kagan J. C. Medzhitov R. (2006). Phosphoinositide-mediated adaptor recruitment controls Toll-like receptor signaling. Cell 125, 943–955. doi: 10.1016/j.cell.2006.03.047 16751103

[B42] Karron R. A. San Mateo J. Wanionek K. Collins P. L. Buchholz U. J. (2018). Evaluation of a live attenuated human metapneumovirus vaccine in adults and children. J. Pediatr. Infect. Dis. Soc. 7, 86–89. doi: 10.1093/jpids/pix006 PMC6075531 28444226

[B43] Kato H. Takeuchi O. Sato S. Yoneyama M. Yamamoto M. Matsui K.. (2006). Differential roles of MDA5 and RIG-I helicases in the recognition of RNA viruses. Nature 441, 101–105. doi: 10.1038/nature04734 16625202

[B44] Kluge S. F. Sauter D. Kirchhoff F. (2015). SnapShot: antiviral restriction factors. Cell 163, 774–774.e1. doi: 10.1016/j.cell.2015.10.019 26496613

[B45] Kolli D. Bao X. Liu T. Hong C. Wang T. Garofalo R. P.. (2011). Human metapneumovirus glycoprotein G inhibits TLR4-dependent signaling in monocyte-derived dendritic cells. J. Immunol. 187, 47–54. doi: 10.4049/jimmunol.1002589 21632720 PMC3119724

[B46] Krusat T. Streckert H. J. (1997). Heparin-dependent attachment of respiratory syncytial virus (RSV) to host cells. Arch. Virol. 142, 1247–1254. doi: 10.1007/s007050050156 9229012

[B47] Kuiken T. van den Hoogen B. G. van Riel D. A. Laman J. D. van Amerongen G. Sprong L.. (2004). Experimental human metapneumovirus infection of cynomolgus macaques (Macaca fascicularis) results in virus replication in ciliated epithelial cells and pneumocytes with associated lesions throughout the respiratory tract. Am. J. Pathol. 164, 1893–1900. doi: 10.1016/S0002-9440(10)63750-9 15161626 PMC1615765

[B48] Lê V. B. Riteau B. Alessi M. C. Couture C. Jandrot-Perrus M. Rhéaume C.. (2018). Protease-activated receptor 1 inhibition protects mice against thrombin-dependent respiratory syncytial virus and human metapneumovirus infections. Br. J. Pharmacol. 175, 388–403. doi: 10.1111/bph.14084 29105740 PMC5758388

[B49] Le Nouën C. Hillyer P. Brock L. G. Winter C. C. Rabin R. L. Collins P. L.. (2014). Human metapneumovirus SH and G glycoproteins inhibit macropinocytosis-mediated entry into human dendritic cells and reduce CD4+ T cell activation. J. Virol. 88, 6453–6469. doi: 10.1128/JVI.03261-13 24672038 PMC4093882

[B50] Liao S. Bao X. Liu T. Lai S. Li K. Garofalo R. P.. (2008). Role of retinoic acid inducible gene-I in human metapneumovirus-induced cellular signalling. J. Gen. Virol. 89, 1978–1986. doi: 10.1099/vir.0.2008/000778-0 18632970 PMC2865242

[B51] Lin B. Ferguson C. White J. T. Wang S. Vessella R. True L. D.. (1999). Prostate-localized and androgen-regulated expression of the membrane-bound serine protease TMPRSS2. Cancer Res. 59, 4180–4184. 10485450

[B52] Liu X. Yu J. Song S. Yue X. Li Q. (2017). Protease-activated receptor-1 (PAR-1): a promising molecular target for cancer. Oncotarget 8, 107334–107345. doi: 10.18632/oncotarget.21015 29291033 PMC5739818

[B53] Loo Y. M. Gale M. Jr. (2011). Immune signaling by RIG-I-like receptors. Immunity 34, 680–692. doi: 10.1016/j.immuni.2011.05.003 21616437 PMC3177755

[B54] Lozach P. Y. Burleigh L. Staropoli I. Amara A. (2007). The C type lectins DC-SIGN and L-SIGN: receptors for viral glycoproteins. Methods Mol. Biol. 379, 51–68. doi: 10.1007/978-1-59745-393-6_4 17502670 PMC7122727

[B55] Lu R. M. Hwang Y. C. Liu I. J. Lee C. C. Tsai H. Z. Li H. J.. (2020). Development of therapeutic antibodies for the treatment of diseases. J. BioMed. Sci. 27, 1. doi: 10.1186/s12929-019-0592-z 31894001 PMC6939334

[B56] Martínez-Espinoza I. Bungwon A. D. Guerrero-Plata A. (2023). Human metapneumovirus-induced host microRNA expression impairs the interferon response in macrophages and epithelial cells. Viruses 15, v15112272. doi: 10.3390/v15112272 PMC10675405 38005948

[B57] McMichael T. M. Zhang Y. Kenney A. D. Zhang L. Zani A. Lu M.. (2018). IFITM3 restricts human metapneumovirus infection. J. Infect. Dis. 218, 1582–1591. doi: 10.1093/infdis/jiy361 29917090 PMC6173576

[B58] Otsuka M. Jing Q. Georgel P. New L. Chen J. Mols J.. (2007). Hypersusceptibility to vesicular stomatitis virus infection in Dicer1-deficient mice is due to impaired miR24 and miR93 expression. Immunity 27, 123–134. doi: 10.1016/j.immuni.2007.05.014 17613256

[B59] Panda S. Mohakud N. K. Pena L. Kumar S. (2014). Human metapneumovirus: review of an important respiratory pathogen. Int. J. Infect. Dis. 25, 45–52. doi: 10.1016/j.ijid.2014.03.1394 24841931 PMC7110553

[B60] Paoloni-Giacobino A. Chen H. Peitsch M. C. Rossier C. Antonarakis S. E. (1997). Cloning of the TMPRSS2 gene, which encodes a novel serine protease with transmembrane, LDLRA, and SRCR domains and maps to 21q22.3. Genomics 44, 309–320. doi: 10.1006/geno.1997.4845 9325052

[B61] Piñana M. González-Sánchez A. Andrés C. Abanto M. Vila J. Esperalba J.. (2023). The emergence, impact, and evolution of human metapneumovirus variants from 2014 to 2021 in Spain. J. Infect. 87, 103–110. doi: 10.1016/j.jinf.2023.05.004 37178807

[B62] Poliseno L. Lanza M. Pandolfi P. P. (2024). Coding, or non-coding, that is the question. Cell Res. 34, 609–629. doi: 10.1038/s41422-024-00975-8 39054345 PMC11369213

[B63] Prelli Bozzo C. Nchioua R. Volcic M. Koepke L. Krüger J. Schütz D.. (2021). IFITM proteins promote SARS-CoV-2 infection and are targets for virus inhibition in *vitro* . Nat. Commun. 12, 4584. doi: 10.1038/s41467-021-24817-y 34321474 PMC8319209

[B64] Ren J. Wang Q. Kolli D. Prusak D. J. Tseng C. T. Chen Z. J.. (2012). Human metapneumovirus M2-2 protein inhibits innate cellular signaling by targeting MAVS. J. Virol. 86, 13049–13061. doi: 10.1128/JVI.01248-12 23015697 PMC3497653

[B65] Schildgen V. van den Hoogen B. Fouchier R. Tripp R. A. Alvarez R. Manoha C.. (2011). Human Metapneumovirus: lessons learned over the first decade. Clin. Microbiol. Rev. 24, 734–754. doi: 10.1128/CMR.00015-11 21976607 PMC3194831

[B66] Seth R. B. Sun L. Chen Z. J. (2006). Antiviral innate immunity pathways. Cell Res. 16, 141–147. doi: 10.1038/sj.cr.7310019 16474426

[B67] Shirogane Y. Takeda M. Iwasaki M. Ishiguro N. Takeuchi H. Nakatsu Y.. (2008). Efficient multiplication of human metapneumovirus in Vero cells expressing the transmembrane serine protease TMPRSS2. J. Virol. 82, 8942–8946. doi: 10.1128/JVI.00676-08 18562527 PMC2519639

[B68] Shukla D. Liu J. Blaiklock P. Shworak N. W. Bai X. Esko J. D.. (1999). A novel role for 3-O-sulfated heparan sulfate in herpes simplex virus 1 entry. Cell 99, 13–22. doi: 10.1016/S0092-8674(00)80058-6 10520990

[B69] Sidhu T. S. French S. L. Hamilton J. R. (2014). Differential signaling by protease-activated receptors: implications for therapeutic targeting. Int. J. Mol. Sci. 15, 6169–6183. doi: 10.3390/ijms15046169 24733067 PMC4013622

[B70] Skalsky R. L. Cullen B. R. (2010). Viruses, microRNAs, and host interactions. Annu. Rev. Microbiol. 64, 123–141. doi: 10.1146/annurev.micro.112408.134243 20477536 PMC3621958

[B71] Smith S. E. Busse D. C. Binter S. Weston S. Diaz Soria C. Laksono B. M.. (2019). Interferon-induced transmembrane protein 1 restricts replication of viruses that enter cells via the plasma membrane. J. Virol. 93, 02003–02018. doi: 10.1128/JVI.02003-18 PMC6401438 30567988

[B72] Soilleux E. J. Barten R. Trowsdale J. (2000). DC-SIGN; a related gene, DC-SIGNR; and CD23 form a cluster on 19p13. J. Immunol. 165, 2937–2942. doi: 10.4049/jimmunol.165.6.2937 10975799

[B73] Soto J. A. Gálvez N. M. S. Benavente F. M. Pizarro-Ortega M. S. Lay M. K. Riedel C.. (2018). Human metapneumovirus: mechanisms and molecular targets used by the virus to avoid the immune system. Front. Immunol. 9, 2466. doi: 10.3389/fimmu.2018.02466 30405642 PMC6207598

[B74] Spann K. M. Loh Z. Lynch J. P. Ullah A. Zhang V. Baturcam E.. (2014). IRF-3, IRF-7, and IPS-1 promote host defense against acute human metapneumovirus infection in neonatal mice. Am. J. Pathol. 184, 1795–1806. doi: 10.1016/j.ajpath.2014.02.026 24726644

[B75] Stewart C. R. Marsh G. A. Jenkins K. A. Gantier M. P. Tizard M. L. Middleton D.. (2013). Promotion of Hendra virus replication by microRNA 146a. J. Virol. 87, 3782–3791. doi: 10.1128/JVI.01342-12 23345523 PMC3624204

[B76] Takada Y. Ye X. Simon S. (2007). The integrins. Genome Biol. 8, 215. doi: 10.1186/gb-2007-8-5-215 17543136 PMC1929136

[B77] Thammawat S. Sadlon T. A. Hallsworth P. G. Gordon D. L. (2008). Role of cellular glycosaminoglycans and charged regions of viral G protein in human metapneumovirus infection. J. Virol. 82, 11767–11774. doi: 10.1128/JVI.01208-08 18786997 PMC2583676

[B78] van den Hoogen B. G. Bestebroer T. M. Osterhaus A. D. Fouchier R. A. (2002). Analysis of the genomic sequence of a human metapneumovirus. Virology 295, 119–132. doi: 10.1006/viro.2001.1355 12033771

[B79] van den Hoogen B. G. de Jong J. C. Groen J. Kuiken T. de Groot R. Fouchier R. A.. (2001). A newly discovered human pneumovirus isolated from young children with respiratory tract disease. Nat. Med. 7, 719–724. doi: 10.1038/89098 11385510 PMC7095854

[B80] Velayutham T. S. Kolli D. Ivanciuc T. Garofalo R. P. Casola A. (2013). Critical role of TLR4 in human metapneumovirus mediated innate immune responses and disease pathogenesis. PloS One 8, e78849. doi: 10.1371/journal.pone.0078849 24205331 PMC3812158

[B81] Weston S. Czieso S. White I. J. Smith S. E. Kellam P. Marsh M. (2014). A membrane topology model for human interferon inducible transmembrane protein 1. PloS One 9, e104341. doi: 10.1371/journal.pone.0104341 25105503 PMC4126714

[B82] Wu W. Choi E. J. Lee I. Lee Y. S. Bao X. (2020). Non-coding RNAs and their role in respiratory syncytial virus (RSV) and human metapneumovirus (hMPV) infections. Viruses 12(3):345. doi: 10.3390/v12030345 32245206 PMC7150941

[B83] Xu Y. Sun F. Chuai Z. Wang J. Bai Z. Bian C.. (2024). Cold-adapted influenza vaccine carrying three repeats of a respiratory syncytial virus (RSV) fusion glycoprotein epitope site protects BALB/c mice and cotton rats against RSV infection. Antiviral Res. 229, 105960. doi: 10.1016/j.antiviral.2024.105960 38986872

[B84] Yim K. C. Cragin R. P. Boukhvalova M. S. Blanco J. C. Hamlin M. E. Boivin G.. (2007). Human metapneumovirus: enhanced pulmonary disease in cotton rats immunized with formalin-inactivated virus vaccine and challenged. Vaccine 25, 5034–5040. doi: 10.1016/j.vaccine.2007.04.075 17543425 PMC1937335

[B85] Zhang W. Zhang L. Zan Y. Du N. Yang Y. Tien P. (2015). Human respiratory syncytial virus infection is inhibited by IFN-induced transmembrane proteins. J. Gen. Virol. 96, 170–182. doi: 10.1099/vir.0.066555-0 25228491

[B86] Zhao H. Feng Q. Feng Z. Zhu Y. Ai J. Xu B.. (2022). Clinical characteristics and molecular epidemiology of human metapneumovirus in children with acute lower respiratory tract infections in China, 2017 to 2019: A multicentre prospective observational study. Virol. Sin. 37, 874–882. doi: 10.1016/j.virs.2022.08.007 36007839 PMC9797368

[B87] Zhou T. Chen Y. Hao L. Zhang Y. (2006). DC-SIGN and immunoregulation. Cell Mol. Immunol. 3, 279–283. 16978536

